# Supplementary material for: The Ithildin library for efficient numerical solution of anisotropic reaction-diffusion problems in excitable media
Source: PLoS One. 2024 Sep 19;19(9):e0303674. doi: 10.1371/journal.pone.0303674 (PMC11412660; doi:10.1371/journal.pone.0303674)
Supplement: S1 Appendix — (PDF) [file pone.0303674.s001.pdf]

Appendix of "The Ithildin library for efficient numerical solution of anisotropic reaction-diffusion problems in excitable media"

Desmond Kabus[1,2], Marie Cloet[1], Christian Zemlin[3], Olivier Bernus[4], Hans Dierckx[1*]

1 Department of Mathematics, KU Leuven Campus Kortrijk (KULAK), Etienne Sabbelaan 53, 8500 Kortrijk, Belgium
2 Laboratory of Experimental Cardiology, Leiden University Medical Center (LUMC), Albinusdreef 2, 2333 ZA Leiden, The Netherlands
3 Division of Cardiothoracic Surgery, Department of Surgery, University of Washington School of Medicine, 660 South Euclid Avenue, 63110 St Louis, MO, United States of America
4 Univ. Bordeaux, Inserm, Centre de Recherche Cardio-Thoracique de Bordeaux U1045, IHU Liryc, Hôpital Xavier Arnozan, Avenue du Haut Lévêque, 33600 Pessac, France

* h.dierckx@kuleuven.be

Appendix

This section contains C++ code to run the four simulations that are used throughout this paper. An overview of their parameters is given in Table 4.

Sim 1: Spiral break-up in the Smooth-Karma model

This is the source code to the Ithildin simulation used as the main example throughout this paper.

```
#include "ithildin.h"

int main(int argc, char** argv){
    Mpiclass mpi(argc, argv);

    // define model
    Model_SmooKa model1;

    // define model for obstacles
    Model_SmooKa model2;
    model2.hidden_eps *= 1.5;

    // rescale time and space
    const float scale_time = 2.4; // ms/t.u.
    const float scale_space = 0.273; // mm/s.u.
    ModelWrapper_RescaleTimeSpace model1a{
        &model1, scale_time, scale_space, 2,
    };
    ModelWrapper_RescaleTimeSpace model2a{
        &model2, scale_time, scale_space, 2,
    };

    // rescale u to voltage, such that: u is in range [0, 1]
```

```
    const float ucrit = 0.5;
    const float ustim = 0.75;
    ModelWrapper_RescaleVars model1b{&model1a, "u", 0.25, 0.};
    ModelWrapper_RescaleVars model2b{&model2a, "u", 0.25, 0.};

    // record diffusion term for EGMs
    ModelWrapper_RecordDiffusion model1c{&model1b};
    model1c.record("u");
    ModelWrapper_RecordDiffusion model2c{&model2b};
    model2c.record("u");

    // record local activation time
    ModelWrapper_RecordActivationTime model1d{&model1c};
    model1d.record("u", ucrit);
    ModelWrapper_RecordActivationTime model2d{&model2c};
    model2d.record("u", ucrit);

    // combine models
    Model_multi model{{&model1d, &model2d}};

    // geometry parameters
    const size_t Nx = 100;
    const size_t Ny = 70;
    const float dx = 0.2;
    const float dy = dx;
    const float Lx = Nx*dx;
    const float Ly = Ny*dy;

    // set up geometry
    vector<int> size{Nx, Ny, 1};
    vector<float> deltas{dx, dy, 1.};
    Geometry_Iso geom{&mpi, size, deltas, &model};
    geom.add_inhom(
            Shape::Sphere(0.25*Ly, {0.7*Lx, 0.3*Ly}), 2
    );
    geom.add_inhom(
            Shape::Rect({0.2*Lx, 0.7*Ly}, {0.3*Lx, 0.8*Ly}), 2
    );
    geom.add_inhom(Shape([&](vector<float> p) {
        return p[1] < 0.1*Ly*(1. + 0.7*sin(14.*p[0]/Ly));
    }, "waves"), 0);

    // set up simulation and timing
    const int Nframe = 100; // number of frames
    const float framedur = 20.; // time between frames
    const float sampledur = 2.; // sampling time for time traces
    Sim sim{framedur, Nframe, &mpi, &model, &geom, "everything", 0};

    // filament tracking
    model.define_tip("u", ucrit, "v", 0.95);
    sim.enable_filament_recording(0, sampledur);

```

```
    // recording temporal data at a specific location
    sim.add_history_point({0.5*Lx, 0.5*Ly});
    sim.sensorlag = sampledur;

    // record EGMs
    sim.add_egm_electrode({0.5*Lx, 0.5*Ly, 1}, sampledur, "diffusu");
    const float lambda = 0.3; // proportionality factor between \
        // intra- and extracellular conduction matrix
    const float beta = 1e5; // 1/m; area-to-volume ratio
    const float capacitance = 1e-2; // F/m²; specific cell \
        // membrane capacitance
    const float conductivity = 0.5; // S/m; lumped conductivity
    for(Egm* egm : sim.egm_collection) {
        egm->construct_kernel(lambda, beta, capacitance, conductivity);
    }

    // set up stimulus protocol
    Source sour(&model, &geom, &mpi, &sim);
    sour.set_val("u", ustim);

    // S0: initial stimulus
    sour.stimulate(Shape::Rect({0, 0}, {0.1*Lx, Ly}));

    // wait until S1S2 protocol starts
    const float timeS1 = 600.; // ms
    sour.schedule.emplace(timeS1, [&]() {
        // S1: first stimulus of S1S2
        sour.stimulate(Shape::Rect({0, 0}, {0.1*Lx, Ly}));

        // S2: second stimulus of S1S2
        sour.setupS2(Point::Phys(&geom, &mpi, 0.5*Lx, 0.9*Ly), 0, ucrit,
            [&]() -> void {
                sour.stimulate(Shape::Rect({0, 0}, {Lx, 0.5*Ly}));
            }
        );
    });

    return sim.run(&sour);
}
```

Sim 2: 2D spiral wave in the AP96 model

This example illustrates the S1S2 protocol in an easy-to-interpret setting.

```
#include "ithildin.h"

int main(int argc, char** argv){
    Mpiclass mpi(argc, argv);

    Model_AP model0(0.15, 0.002, 8, 0.2, 0.3, 20);

    // rescale time and space [@aliev1996simple]
    const float scale_time = 12.9; // ms/t.u.
```

```
    const float scale_space = 1.; // mm/s.u.
    ModelWrapper_RescaleTimeSpace model{
        &model0, scale_time, scale_space, 2,
    };

    vector<int> size = {120, 120, 1};
    vector<float> dx = {1.0, 1.0, 1.0};
    float Lx = size[0]*dx[0];
    float Ly = size[1]*dx[1];
    Geometry_Iso geom{&mpi, size, dx, &model};

    float framedur = 1.*scale_time;
    int Nframe = 65;
    Sim sim{framedur, Nframe, &mpi, &model, &geom, "s1s2", 0};
    sim.enable_filament_recording(0, framedur);

    Source sour{&model, &geom, &mpi, &sim};
    sour.set_val("u", 0.75);

    // S1
    sour.stimulate(Shape::Rect({0, 0}, {0.1f*Lx, Ly}));

    // S2
    const Point sensor = Point::Phys(&geom, &mpi, 0.6f*Lx, 0.5f*Ly, 0);
    LOG("sensor", sensor.get_phys());
    sour.setupS2(sensor, 0, 0.5, [&]() -> void {
        sour.stimulate(Shape::Rect({0, 0}, {Lx, 0.2f*Ly}));
    });

    return sim.run(&sour);
}
```

Sim 3: 2D spiral wave in the BOCF model

This simulation is used to get an initial state for a 3D simulation in ventricular geometry in the following example.

```
#include "ithildin.h"

int main(int argc, char** argv){
    Mpiclass mpi(argc, argv);

    // define model
    Model_BO modelbo{1};
    const float ucrit = 0.5;
    const float ustim = 1.0;

    // record local activation time
    ModelWrapper_RecordActivationTime model{&modelbo};
    model.record("u", ucrit);

    // set up geometry
    vector<int> size{450, 450, 1};
```

```
    vector<float> dx{0.3, 0.3, 1.};
    const float Lx = size[0]*dx[0];
    const float Ly = size[1]*dx[1];
    Geometry_Iso geom = Geometry_Iso(&mpi, size, dx, &model);

    // set up simulation and timing
    const float framedur = 20.;
    const int Nframe = 34;
    Sim sim{framedur, Nframe, &mpi, &model, &geom, "bocf2d", 0};

    // set up stimulus protocol
    Source sour(&model, &geom, &mpi, &sim);
    sour.set_val("u", ustim);

    // S1: first stimulus of S1S2
    sour.stimulate(Shape::Rect({0, 0}, {0.1*Lx, Ly}));

    // S2: second stimulus of S1S2
    const Point sensor = Point::Phys(&geom, &mpi, 0.75*Lx, 0.5*Ly);
    LOG("sensor", sensor.get_phys());
    sour.setupS2(sensor, 0, ucrit, [&]() -> void {
        sour.stimulate(Shape::Rect({0, 0}, {0.75*Lx, 0.75*Ly}));
    });

    return sim.run(&sour);
}
```

Sim 4: 3D spiral wave in the BOCF model

This 3D spiral wave is stimulated by extending a 2D spiral wave and placing it on ventricular geometry.

```
#include "ithildin.h"

int main(int argc, char** argv){
    Mpiclass mpi(argc, argv);

    // choose model
    Model_BO modelbo{1};
    const float ucrit = 0.5;
    const float ustim = 1.0;

    // record local activation time
    ModelWrapper_RecordActivationTime model{&modelbo};
    model.record("u", ucrit);

    // set up geometry
    vector<float> Ds {1, 0.25, 0.25};
    vector<int> size {168, 208, 231};
    vector<float> deltas {0.43, 0.43, 0.5};
    Geometry_OrtAniso geom{
        size, deltas, "ventricles", &model, Ds, &mpi,
    };
```

```cpp

    // set up simulation
    float framedur = 20.;
    int Nframe = 30;
    Sim sim{framedur, Nframe, &mpi, &model, &geom, "bocf3d", 0};

    // filament tracking
    sim.enable_filament_recording(0, framedur);

    // set up stimuli
    Source sour{&model, &geom, &mpi, &sim};

    // transform 2D simulation onto 3D ventricles, PD on RV
    NDArray<float> transform; transform.read("bocf2d3d.transform.npy");
    NDArray<float> vars = sour.read_from_stem("bocf2d_0", 34);
    const vector<size_t> shape{
        vars.get_shape(0), size[0], size[1], size[2],
    };
    vars = vars.interpolate(&mpi, shape,
        [&transform](std::vector<float> x){
            if(x.size() != 4) { ERROR("invalid shape"); }
            std::vector<float> z{1., x[1], x[2], x[3]};
            std::vector<float> y(4, 0);
            for(size_t i=0; i<4; i++) {
                for(size_t j=0; j<4; j++) {
                    y[i] += transform(i,j)*z[j];
                }
            }
            return std::vector<float>{x[0], y[1], y[2], y[3]};
        }
    );
    sour.set_frame(vars);

    return sim.run(&sour);
}
```

Sim 5: Cardiac electrophysiology benchmark

This is the benchmark problem as proposed by Niederer *et al.* (2011) [2] using the cell model by Ten Tusscher and Panfilov (2006) [1].

```cpp
#include "ithildin.h"

int main(int argc, char** argv){
    std::vector<float> arg = strings_to_floats(argc, argv);
    Mpiclass mpi(argc, argv);

    // arg 1: serial number
    const int serialnr = (arg.size() > 1) ? round(arg.at(1)) : 0;
    MASTER_INFO("serialnr", serialnr);

    // arg 2: discretization {0.1, 0.2, 0.5}
    const float dx = (arg.size() > 2) ? arg.at(2) : 0.2; // mm
```

```
    // arg 3: PDE time steps {0.005, 0.01, 0.05}
    const float dt = (arg.size() > 3) ? arg.at(3) : 0.05; // ms

    // define model
    Model_TP06 model_tp06;
    ModelWrapper_RecordActivationTime model{&model_tp06};
    model.record("V", 0);

    // constants
    const float C_m = 0.01; // µF/mm² (membrane capacitance)
    const float chi = 140; // 1/mm (surface-to-volume ratio)

    // diffusivities intra/extra longitudinal/transversal
    const float scale = 1 / (chi * C_m); // mm³/µF
    const float Dil = scale*0.17; // mm²/ms
    const float Dit = scale*0.019; // mm²/ms
    const float Del = scale*0.62; // mm²/ms
    const float Det = scale*0.24; // mm²/ms

    // diffusivities longitudinal/transversal
    const float Dl = Dil*Del / (Dil+Del); // mm²/ms
    const float Dt = Dit*Det / (Dit+Det); // mm²/ms

    // diffusivity tensor only scales, Pmat stores magnitude
    vector<float> D{1., Dt/Dl, Dt/Dl}; // 1
    model.set_Pmat(Dl); // mm²/ms

    // domain size
    const float Lx = 20; // mm
    const float Ly = 7; // mm
    const float Lz = 3; // mm

    // set up geometry
    vector<float> deltas{dx, dx, dx};
    vector<int> size{
        int(Lx/deltas[0])+1, int(Ly/deltas[1])+1, int(Lz/deltas[2])+1,
    };
    vector<float> tissue_angles{0., 0.};
    vector<float> diffpars{1, 1, 0};
    Geometry_OrtAniso geom{
        size, deltas, &model, tissue_angles, diffpars, D, &mpi,
    };

    // limit output
    for(size_t ivar=0; ivar<model.get_Nvar(); ivar++)
    { geom.set_bwritevar(ivar, 0); }
    geom.set_bwritevar(model.get_ivar("V"), 1);
    geom.set_bwritevar(model.get_ivar("latV"), 1);

    // set up simulation and timing
    const int Nframe = 15; // number of frames
```

```
    const float framedur = 10.; // time between frames in ms
    Sim sim{
        dt, framedur, Nframe, 0, &mpi, &model, &geom,
        "niederer2011benchmark", serialnr, false,
    };

    // set up stimulus
    Source sour(&model, &geom, &mpi, &sim);
    const float I_stim = 50; // mA/cm³ (stimulus current)
    const float i_stim = I_stim * scale; // A/F = V/s = mV/ms
    sour.set_val("V", i_stim); // mV/ms
    sour.stimulate({
            Shape::Rect({0, 0, 0}, {1.5, 1.5, 1.5}),
            1.0, // amplitude factor
            2.0, // ms; duration
            true, // flag: additive stimulus
    });

    return sim.run(&sour);
}
```

References

1. Ten Tusscher KH, Panfilov AV. Alternans and spiral breakup in a human ventricular tissue model. American Journal of Physiology-Heart and Circulatory Physiology. 2006;291(3):H1088–H1100. doi:10.1152/ajpheart.00109.2006.
2. Niederer SA, Kerfoot E, Benson AP, Bernabeu MO, Bernus O, Bradley C, et al. Verification of Cardiac Tissue Electrophysiology Simulators Using an N-version Benchmark. Philosophical Transactions of the Royal Society A: Mathematical, Physical and Engineering Sciences. 2011;369(1954):4331–4351. doi:10.1098/rsta.2011.0139.
